# Urinary incontinence after uncomplicated spontaneous vaginal birth in primiparous women during the first year after birth

**DOI:** 10.1007/s00192-019-03975-0

**Published:** 2019-05-28

**Authors:** Susanne Åhlund, Emilia Rothstein, Ingela Rådestad, Sofia Zwedberg, Helena Lindgren

**Affiliations:** 1 Department of Women’s and Children’s Health, Karolinska Institutet, Tomtebodavägen 18A, Solna, 17177 Stockholm, Sweden; 2 Department of Clinical Science, Intervention and Technology, Karolinska Institutet, Stockholm, Sweden; 3 Department of Benign Gynaecology and Reproductive Medicine, Karolinska University Hospital Huddinge, Stockholm, Sweden; 4 Department of Health-promoting Science, Sophiahemmet University, Stockholm, Sweden; 5 Department of Obstetrics, Karolinska University Hospital Solna, Stockholm, Sweden

**Keywords:** Urinary incontinence, Postpartum, Primiparous, Vaginal, Birth

## Abstract

**Introduction and hypothesis:**

Urinary incontinence (UI) is associated with pregnancy and parity and can cause health problems for women. Our objective was to explore risk factors for UI and its effect on women’s daily activities, psychological health and wellbeing 9–12 months postpartum in a low-risk primiparous population.

**Methods:**

In this prospective cohort study, first-time mothers in a low-risk population with a spontaneous vaginal birth reported the occurrence of UI and its effect on daily activities and on their psychological health and wellbeing in a questionnaire completed 1 year after birth. Descriptive and comparative statistics were employed for the analysis.

**Results:**

A total of 410 women (75.7%) completed the questionnaire. The self-reported rates of stress urinary incontinence, urge urinary incontinence and mixed urinary incontinence were 45.4%, 38.0% and 27.0% respectively. Neither the duration of the second stage of labour, the baby’s head circumference or its birth weight were associated with the incidence of UI. There was an association between reported negative impact on daily activities and more negative psychological wellbeing (*p* < 0.001).

**Conclusions:**

Urinary incontinence was common among primiparous women at 9–12 months postpartum. Women whose symptoms had a negative impact on their daily activities reported more psychological suffering.

## Introduction

Urinary incontinence (UI) has been reported to have a strong association with vaginal birth, and can cause health problems for women [1, 2]. Stress urinary incontinence (SUI) and urge urinary incontinence (UUI) have been reported to be more common after vaginal birth than after caesarean section, although the differences decrease with time after giving birth [3].

Conflicting findings have been reported concerning risk factors for the occurrence of UI after vaginal birth. The most important risk factors for UI have been identified as body mass index (BMI) >30, advanced age, oxytocin use, prolonged second stage of labour, baby’s birth weight > 4,000 g and large head circumference [4]. The most important obstetric risk factor for UI among primiparous women is instrumental vaginal birth; a review states that 32% of this group report UI at 3 months postpartum [1].

Definition used in this study is according to the joint report from the International Continence Society (ICS) and the International Urogynecological Association (IUGA) [5]. Longitudinal data on UI, and on different types of UI 1 year postpartum in primiparous women after vaginal birth, remain sparse. Studies of UI among primiparous women indicate considerable variation in prevalence, mainly due to differences in study design, study population, type of UI and different methods of assessing UI. A systematic review by Tahtinen et al. reported an SUI prevalence in primiparous women varying from 9% to 68%, whereas the corresponding figure for UUI varied from 8% to 27% after the first year postpartum [6].

In a cohort study by Mannion et al. including 1,574 women, 17% reported that their UI symptoms were associated with a moderate to severe impairment to perform daily tasks 12 months postpartum [7]. Women often perceive UI to be a social stigma, while nevertheless regarding it as a normal consequence of childbearing, which can lead to under-reporting of symptoms [8]. Little is known about whether management of the second stage of labour or neonatal parameters have an impact on the outcome 9–12 months after the birth of the first child. The aim of this study was to investigate the prevalence and effect of UI and its impact on women’s daily activities, in addition to its impact on psychological health and wellbeing, 9–12 months postpartum in a low-risk primiparous population.

## Materials and methods

This prospective cohort study was conducted among primiparous women who had given birth at one of two delivery wards in Stockholm between 1 November 2013 and 16 February 2015. One delivery ward provides care to approximately 4,100 women/year, and the other to 6,500 women/year. Both delivery wards handle both high- and low-risk pregnancies.

The study included primiparous, Swedish-speaking women, with spontaneous onset or induction of labour, who gave birth at gestational age ≥ 37 + 0 weeks. Women with diabetes mellitus (gestational or manifest), female genital mutilation, intrauterine growth restriction, stillbirth, breech presentation, instrumental birth, multiple pregnancy or pregnancy again within 1 year postpartum were excluded. Women who met the inclusion criteria were asked to participate on admission to the delivery ward.

Data collection for this study started in November 2014 and was finished in the spring of 2016. A postal questionnaire covering various persisting symptoms of pelvic floor dysfunction was sent to all women included in the study 12 months after birth. UI, defined according to IUGA and ICS [5], was one of the pelvic floor dysfunctions covered by the questionnaire, which contained the UDI-6 and the UIQ-7 [9]. The questions that concern the occurrence of UI are presented in this study. The questionnaire also included questions about sociodemographic background: age, level of education, marital status, BMI and tobacco use. Face-to-face validation of the questionnaire had been conducted with 12 women, after which some minor changes had been made.

The primary outcome was women’s self-reported occurrence of UI 9–12 months after giving birth. The impact of UI experienced by women on their daily activities and psychological well-being were secondary outcomes.

The variables documented by the midwives after the birth in the study-specific protocol were time of complete cervical dilatation, use of oxytocin for labour augmentation, presentation, birth position and degree of perineal injury [10]. BMI during the first trimester was retrieved from the hospital medical records. Obstetric variables, such as onset of labour, epidural analgesia, respective onset times of passive and active second stages and time of birth, were retrieved from the hospital medical records. Onset of labour was noted as either spontaneous or induced. Neonatal variables recorded from the hospital medical records were head circumference and birth weight. Continuous variables categorised were BMI (<18.5, 18.5–24.9, 25.0–29.9, >30), age (<25, 25–35, >35) and the baby’s birth weight (<3,000 g, 3,000–3,499 g, 3,500–4,000 g, >4,000 g) and head circumference (<34.9 cm, >35 cm).

The passive second stage was defined and calculated as the interval between being fully dilated cervix and the onset of the active phase, whereas the active second stage was defined and calculated as the onset of active pushing to the time of birth [11]. The passive second stage was categorised as >1 h, 1- to <2 h, 2 to <3 h, > 3 h, and the active second stage was categorised as < 30 min, 30–60 min and > 60 min. Birth positions were dichotomized into either flexible sacrum position or non-flexible sacrum position [10] Flexible sacrum position is defined as a birth position that allows flexibility in the sacro-iliac joints, when weight is taken off the sacrum and allows the pelvic outlet to expand [12]. Birth positions with a flexible sacrum position are standing, kneeling, on all-fours, on the birth seat or a lateral position. Birth positions with a non-flexible sacrum are supine position and a semi-recumbent position (see Table 2).

A variable was created to analyse the primary outcome, in which the occurrence of any UI (yes/no) was compared with the length of the second stage of labour. A prolonged second stage was defined according to ACOG guidelines, as >3 h in primiparous women with epidural analgesia, and > 2 h in primiparous woman without epidural analgesia [13] (see Table 3). UI symptoms were classified using the standardised IUGA/ICS terminology. Responding “yes” to both of the items “Do you usually experience urinary leakage related to coughing, sneezing or laughing?” and “Do you usually experience urinary leakage, together with a strong sensation of needing to go to the bathroom?” was classified as having mixed urinary incontinence (MUI) [5].

For the secondary outcomes, UIQ-7 short form responses on UI and its impact on women’s daily activities, relationships and psychological wellbeing were analysed both separately (see Table 4) and as two composite outcomes “Impact on daily activities” and “Psychological wellbeing”. “Impact on daily activities” was defined as answering questions 1–5 with at least “A little bit”. “Psychological non-wellbeing” was defined as answering either of the questions 6 or 7 with at least “A little bit” (see Table 4).

### Statistical analyses

Descriptive statistics (*n*, percentage, median and mean) and Pearson’s Chi-squared test were used to present background characteristics and for analyses of the associations between the categorical variables. Independent samples *t* tests were used to compare means for birth weight and head circumference and the occurrence of UI. *p* values equal to or lower than 0.05 were considered statistically significant. IBM SPSS Statistics for Windows (version 24.0; SPSS, Chicago, IL, USA) was employed for the data analysis.

### Ethics

The study was approved by the Regional Ethics Review Board at Karolinska Institutet (Dnr: 2013/859-31/2).

The informed consent included the women’s permission for the researchers to obtain additional data (background variables) from their antenatal and hospital medical records. All women participating in the study gave written consent. They were informed that they could withdraw from the study at any time without any consequences for their care.

## Results

In total, 541 women were included in this cohort study, while 410 completed the questionnaire 1 year after birth, corresponding to a response rate of 75.7%. The median age was 31.0, and the median BMI was 23.0. Nearly all women (96.3%) were married or cohabiting, almost three out of four (71.2%) had a university or college degree and almost all were non-smokers (Table 1). The obstetric variables are presented in Table 2.

**Table 1:** Socio-demographic background among 410 women in Sweden, 1 year postpartum (*N* = 410)

	*n* (%)
Age	
<25	46 (11.2)
25–35	312 (76.1)
>35	52 (12.7)
Civil status	
Married/cohabiting	395 (96.3)
Single or other	11 (2.7)
Missing	4 (1.0)
Tobacco use	
Yes	14 (3.4)
No	395 (96.4)
Missing	1 (0.2)
BMI the first trimester	
<18.5	14 (3.4)
18.5–24.9	283 (69.0)
25.0–29.9	66 (16.1)
>30.0	14 (3.4)
Missing	33 (8.0)
BMI 12 months postpartum	
<18.5	16 (3.9)
18.5–24.9	299 (72.9)
25.0–29.9	67 (16.3)
>30.0	21 (5.1)
Missing	7 (1.7)
Health-related problems during pregnancy^a^	
Yes	47 (11.5)
No	342 (83.4)
Missing	21 (5.1)

*BMI* body mass index

^a^Composite variables including asthma, thrombosis, chronic kidney disease, endocrine diseases, epilepsy, chronic hypertension

**Table 2:** Obstetric variables among 410 Swedish first-time mothers

	*n* (%)
Induction of labour	
Yes	61 (14.9)
No	348 (84.9)
Missing	1 (0.2)
Epidural analgesia	
Yes	232 (56.6)
No	177 (43.2)
Missing	1 (0.2)
Passive second stage	
<1 h	99 (24.1)
1 to <2 h	195 (47.6)
2 to <3 h	59 (14.4)
> 3 h	37 (9.0)
Missing	20 (4.9)
Active second stage	
<30 min	353 (86.1)
30–60 min	24 (5.9)
>60 min	13 (3.2)
Missing	20 (4.9)
Spontaneous pushing	
Yes	343 (83.7)
No	57 (13.9)
Missing	10 (2.7)
Birth position	
Flexible sacrum position	253 (61.8)
Non-flexible sacrum position	156 (38.0)
Missing	1 (0.2)
Presentation	
Occiput anterior	396 (96.6)
Occiput posterior	14 (3.4)
Birth weight	
<3,000 g	49 (12.0)
3,000–3,499 g	169 (41.2)
3,500–4,000 g	150 (36.6)
>4,000 g	41 (10.0)
Missing	1 (0.2)
Head circumference	
<34.9 cm	278 (67.8)
>35 cm	129 (31.5)
Missing	3 (0.7)
Perineal tear^a^	
Minor (0–1)	85 (20.7)
Medium I (2a+b)	277 (67.6)
Medium II (2c)^b^	30 (7.3)
Severe (3 + 4)	15 (3.7)
Missing	3 (0.7)

^a^Classified according to National Guidelines [14]

^b^Episiotomy (2, 6%) was classified as second-degree tear 2c

The prevalence of UI, based on the UDI-6 questionnaire, was 45.4% (*n* = 186) for SUI, 38.0% (*n* = 156) for UUI and 27.0% (*n* = 110) for MUI. The women who did not report symptoms of UI constituted 43.1% (*n* = 176). Half of the women reported that their symptoms caused mild inconvenience, and 1 in 5 reported severe discomfort (Table 3). There were no differences regarding the sociodemographic variables age, BMI, health-related problems during pregnancy, level of education (Table 1), or obstetric variables (Table 2). We found no statistically significant association between UI and a long second stage of labour (Table 3). The baby’s birth weight (mean 3,491 g, range 2,485–4,970 g) and head circumference (mean 34.7 cm, range 30.5–38.5 cm) had no impact on urgency (*p* = 0.673 and *p* = 0.263 respectively), experience of urinary leakage (*p* = 0.657 and *p* = 0.133 respectively) or experience of leakage when coughing, sneezing or laughing (*p* = 0.556 and *p* = 0.866 respectively). Furthermore, birth weight and head circumference did not have an impact on bladder-emptying problems (*p* = 0.134 and *p* = 0.633 respectively).

**Table 3:** Urinary incontinence among 410 Swedish first-time mothers 9–12 months postpartum

	Total (*N* = 410) *n* (%)	No prolonged second stage *n* = 349	Prolonged second stage *n* = 41	*p* value
Do you usually need to go to the bathroom to pee often?				
Yes	169 (41.3)	146 (42.2)	13 (31.7)	0.19
No	238 (58.0)	200 (57.8)	28 (68.3)	
Missing	3 (0.7)			
If yes, how much does it bother you?				
A lot	13 (7.7)			
A little bit	98 (58.0)			
Not at all	37 (21.9)			
Missing	21 (12.4)			
Do you usually have urinary leakage, together with a strong sensation of needing to go to the bathroom?				
Yes	156(38.0)	131 (37.8)	17 (41.5)	0.64
No	252(61.5)	216 (62.2)	24 (58.5)	
Missing	2 (0.5)			
If yes, how much does it bother you?				
A lot	33 (21.2)			
A little bit	92 (59.0)			
Not at all	11 (7.1)			
Missing	20 (12.7)			
Do you usually have urinary leakage related to coughing, sneezing or laughing?				
Yes	186(45.4)	157 (45.2)	17 (41.5)	0.64
No	222(54.1)	190 (54.8)	24 (58.5)	
Missing	2 (0.5)			
If yes, how much does it bother you?				
A lot	38 (20.4)			
A little bit	102(54.8)			
Not at all	15 (8.1)			
Missing	31 (16.7)			
Do you usually leak small amounts of urine (i.e. a few drops)?				
Yes	137(33.4)	113 (32.7)	17 (42.5)	0.21
No	269(65.6)	233 (67.3)	23 (57.5)	
Missing	4 (1.0)			
If yes, how much does it bother you?				
A lot	27 (19.7)			
A little bit	79 (57.7)			
Not at all	14 (10.2)			
Missing	17 (12.4)			
Do you usually have trouble emptying your bladder?				
Yes	59 (14.4)	53 (15.3)	4 (9.8)	0.34
No	348(84.9)	293 (84.7)	37 (90.2)	
Missing	3 (0.7)			
If yes, how much does it bother you?				
A lot	10 (16.9)			
A little bit	34 (57.6)			
Not at all	5 (8.5)			
Missing	10 (16.9)			

A majority of the women (*n* = 300, 73.2%) reported that their UI symptoms had no impact on their daily activities, relationships or mental health (Table 4). However, almost a third (32.1%) of the women who reported that their symptoms had a negative impact on their daily activities reported a low level of psychological wellbeing during the last 3 months, compared with only 5% of the women who reported that UI had no impact on their daily activities (*p* < 0.001).

**Table 4:** Symptoms of urinary incontinence and impact on daily activities, relationships and mental health in 410 Swedish first-time mothers 9–12 months postpartum

How do the symptoms or conditions related to the bladder or urine usually affect the following:	*n* (%)
1. Ability to do household chores (cooking, cleaning, laundry)?^a^	
Not at all	392 (95.5)
A little bit	13 (3.3)
A lot	1 (0.2)
Missing	4 (1.0)
2. Ability to do physical activities such as walking, swimming, or other exercise?^a^	
Not at all	319 (77.8)
A little bit	73 (17.8)
A lot	14 (3.4)
Missing	4 (1.0)
3. Ability to enjoy entertainment such as going to a movie or concert?^a^	
Not at all	382 (93.2)
A little bit	19 (4.6)
A lot	1 (0.2)
Missing	8 (2.0)
4. Ability to travel by car or bus for more than 30 min from home?^a^	
Not at all	380 (92.7)
A little bit	19 (4.6)
A lot	4 (1.0)
Missing	7 (1.7)
5. Participation in social activities outside your home?^a^	
Not at all	385 (93.9)
A little bit	16 (3.9)
A lot	2 (0.4)
Missing	7 (1.7)
6. Psychological wellbeing (nervousness, depression etc.)?^b^	
Not at all	371 (90.5)
A little bit	28 (6.8)
A lot	6 (1.5)
Missing	5 (1.2)
7. Feeling frustrated?^b^	
Not at all	367 (89.5)
A little bit	27 (6.7)
A lot	11 (2.7)
Missing	5 (1.1)

^a^Included in the composite variable “Impact on daily activities”

^b^Included in the composite variable “Psychological wellbeing”

## Discussion

In this cohort study including low-risk primiparous women who had undergone vaginal birth, we found that about 40% had symptoms of UI, of which SUI was the most common type, 9–12 months after birth. Obstetric variables, such as duration of the second stage and the baby’s head circumference and birth weight, were not associated with the incidence of UI. The women whose UI affected their daily activities reported significantly impaired psychological wellbeing.

Almost 4 out of 10 women reported UI symptoms, a high prevalence compared with the results of other research studies [15, 16]. This discrepancy may be partly explained by the different definitions of UI and different study designs [1]. In a cohort study by Brown et al., 46.9% of 1,507 primiparous women reported UI within the first 12 months postpartum [17]. A review by Press et al. found that caesarean section was not protective, as the prevalence of SUI and UUI was equivalent, regardless of the mode of birth in studies with follow-up longer than 1 year [18].

The most important risk factor for UI 1 year after vaginal birth has been shown to be UI symptoms before and during pregnancy [15, 19]. In a study by Daly et al., the prevalence of any kind of UI was 38.4% pre-pregnancy and 38.8% during pregnancy; UI prevalence was statistically significantly associated with being obese (BMI >30) [20]. In a study by Gartland et al., 1 in 4 women reported persistent UI during the first 4–18 months postpartum, and 79% of these had had UI during pregnancy [21]. Weakened support of the bladder neck and urethra, together with the hormonal changes during pregnancy, contribute to altered tissue structure, leading to changes in pelvic floor structure tone [22]. The results of this study support the assumption that UI postpartum may be a consequence of the pregnancy itself, resulting from neuromuscular injury and/or loss of urethral and bladder support, rather than of the circumstances related to birth.

Our results are in line with the findings from a number of previous studies; the obstetric variables prolonged the second stage of labour [23, 24], and perineal injury [25, 26], birth weight [15, 23, 24], and head circumference [25] seem to be of no or minor importance for the incidence of UI postpartum. Although Wesnes et al. reported that neonatal parameters such as birth weight > 3,540 g and head circumference > 36 cm may act in synergy to increase the risk of UI at 6 months postpartum in women undergoing spontaneous, non-instrumental vaginal birth [27]. This study did not confirm this finding. The study population, consisting of low-risk women with few interventions, high levels of spontaneous pushing and flexible sacrum position, may explain our outcomes.

About half of the women in this study who had UI reported that their symptoms caused mild inconvenience, and 1 in 5 reported that their symptoms caused severe discomfort. In a study by Brown et al., three-quarters of the women who reported UI at 12 months postpartum regarded their symptoms as a minor problem [17]. Women often perceive UI as something normal and inevitable associated with pregnancy and childbirth, and may describe their problems as secondary to the baby’s health and well-being [28, 29]. In this study, the impact of Ul on women’s daily activities was associated with impaired psychological wellbeing. This finding concurs with those of other studies showing that UI is associated with impaired quality of life and depressive symptoms [30, 31]. Women described changing their routines and planning various activities and social and intimate situations carefully, to avoid embarrassment [7]. As UI can have a negative impact on women’s psychological wellbeing, it is important that they are encouraged to seek health care if it occurs. It should be acknowledged that this is not an inevitable and acceptable consequence of childbirth [8]. Women with UI symptoms do not receive adequate care during the first year after birth; the women did not recall being asked or having discussed UI, despite frequent contact with the health care system [17], and despite this period providing a window of opportunity for early diagnosis and thus for promoting women’s health.

The strengths of the study are the use of validated instruments in assessing UI and a relatively large sample size of primiparous women, who were at a low risk during pregnancy and underwent spontaneous vaginal birth. Furthermore, the detailed data regarding the onset of birth encompasses and strengthens the results. Data were drawn from an earlier prospective cohort study with an experimental design, and a detailed study questionnaire was completed by the midwife assisting the birth [10]. The data provided a full picture of the management and process of labour and birth, particularly the duration of the second stage. The study found that there was a statistically significant difference between responders and non-responders with regard to age and smoking habits; the non-responders were generally younger and smokers. There were no statistically significant differences between women who had completed the questionnaire and those who did not regarding BMI, the severity of perineal injury, the duration of the second stage, the birth position or the baby’s birth weight and head circumference.

A number of limitations in this study may have influenced the results. The main limitation is the lack of information regarding the women’s health, pre-pregnancy history of UI and occurrence of UI during pregnancy. Other limitations were that the study did not use a refined questionnaire that also encompassed the impact of UI on women’s psychological well-being, and that the study sample was recruited from two delivery wards in Stockholm and is not entirely representative of a national sample. Asking women to recall symptoms is subject to bias, and may have led to over- or underestimation of UI. Information on pelvic floor exercises would have been interesting, to investigate any associations with UI. Nevertheless, our findings are similar to those of other comparable studies, showing that the impact of UI on daily activities affects women’s psychological wellbeing.

## Conclusion

In this study, 4 out of 10 women suffered from UI 9–12 months postpartum. Women who experienced a negative impact on their activities as a result of their UI were also at a higher risk of reporting impaired psychological wellbeing. The obstetric variables studied, such as duration of the second stage and the baby’s head circumference and birth weight, were not associated with the prevalence of UI 9–12 months postpartum in this primiparous, low-risk population.

## Abbreviations

UI: Urinary incontinence
SUI: Stress urinary incontinence
UUI: Urge urinary incontinence
MUI: Mixed urinary incontinence
